# Supplementary material for: A 3D brain unit model to further improve prediction of local drug distribution within the brain
Source: PLoS One. 2020 Sep 23;15(9):e0238397. doi: 10.1371/journal.pone.0238397 (PMC7511021; doi:10.1371/journal.pone.0238397)
Supplement: S1 Appendix — (PDF) [file pone.0238397.s001.pdf]

S1 Appendix - Nondimensionalization of the model

We can make Eq (2-16) dimensionless by introducing a change of variables. Here, the original variables are scaled to dimensionless variables by scaling with a characteristic, dimensional scale. We set:

$$
\begin{aligned}
t &= t_c\tau & D^* &= D_c d & k_{1\text{on}} &= k_{1\text{on}_\text{c}} K_{1\text{on}} \\
x &= x_c\xi, & v &= v_c V & k_{1\text{off}} &= k_{1\text{off}_\text{c}} K_{1\text{off}} \\
y &= y_c\eta & C_\text{pl} &= C_{\text{pl}_\text{c}} w & k_{2\text{on}} &= k_{2\text{on}_\text{c}} K_{2\text{on}} \\
z &= z_c\zeta & C_\text{ECF} &= C_\text{c} u & k_{2\text{off}} &= k_{2\text{off}_\text{c}} K_{2\text{off}} \\
B_1 &= B_{1_\text{c}} b_1 & B_1^\text{max} &= B_{1_\text{c}}^\text{max} b_1^\text{max} & P &= P_c p \\
B_2 &= B_{2_\text{c}} b_2 & B_2^\text{max} &= B_{2_\text{c}}^\text{max} b_2^\text{max} & SA_\text{BBB} &= SA_{\text{BBB}_\text{c}} sa_\text{BBB} \\
T_\text{m} &= T_{\text{m}_\text{c}} t_\text{m} & K_\text{m} &= K_{\text{m}_\text{c}} k_\text{m} & v_\text{blood} &= v_{\text{blood}_\text{c}} V_\text{blood}
\end{aligned}
$$

where

$$
\begin{aligned}
t_c &= 1s & D_c &= 10^{-10}\ \text{m}^2\text{s}^{-1} & k_{1\text{on}_\text{c}} &= (\mu\text{mol L}^{-1}\ \text{s})^{-1} \\
x_c &= 10^{-6}\ \text{m} & v_c &= 10^{-6}\ \text{m s}^{-1} & k_{1\text{off}_\text{c}} &= 10^{-2}\ \text{s}^{-1} \\
y_c &= 10^{-6}\ \text{m} & C_{\text{pl}_\text{c}} &= \mu\text{mol L}^{-1} & k_{2\text{on}_\text{c}} &= 10^{-2}\ (\mu\text{mol L}^{-1}\text{s})^{-1} \\
z_c &= 10^{-6}\ \text{m} & C_\text{c} &= \mu\text{mol L}^{-1} & k_{2\text{off}_\text{c}} &= \text{s}^{-1} \\
B_{1_\text{c}} &= \mu\text{mol L}^{-1} & B_{2_\text{c}}^\text{max} &= \mu\text{mol L}^{-1} & P_c &= 10^{-7}\ \text{m s}^{-1} \\
B_{2_\text{c}} &= \mu\text{mol L}^{-1} & B_{2_\text{c}}^\text{max} &= \mu\text{mol L}^{-1} & SA_{\text{BBB}_\text{c}} &= 10^{-6}\ \text{L m}^{-1} \\
T_{\text{m}_\text{c}} &= 10^{-7}\mu\text{mol s}^{-1} & K_{\text{m}_\text{c}} &= 10^2\ \mu\text{mol L}^{-1} & v_{\text{blood}_\text{c}} &= 10^{-3}\ \text{m s}^{-1}
\end{aligned}
$$

This leads to the following dimensionless equation for drug in the blood plasma (example based on Eq (2), but similar for Eq (3)-(4)):

$$
\frac{\partial w}{\partial \tau} = 10^3 V_\text{blood} \frac{\partial w}{\partial \xi}
$$

, and the following system of dimensionless equations for drug within the brain ECF

S1 Fig. The PK in log-scale of unbound drug in the brain ECF ($C_{\mathbf{ECF}}$) compared to the concentration of unbound drug in the blood plasma ($C_{\mathbf{pl}}$, red curve). The transcellular passive permeability, P_{trans}, is set to $0.01 \cdot 10^{-7}$ m s^{-1} (left) and $1 \cdot 10^{-7}$ m s^{-1} (right), while the paracellular permeability, P_{para} is changed from 0 to $1 \cdot 10^{-1}$ m s^{-1} as depicted by different colours.

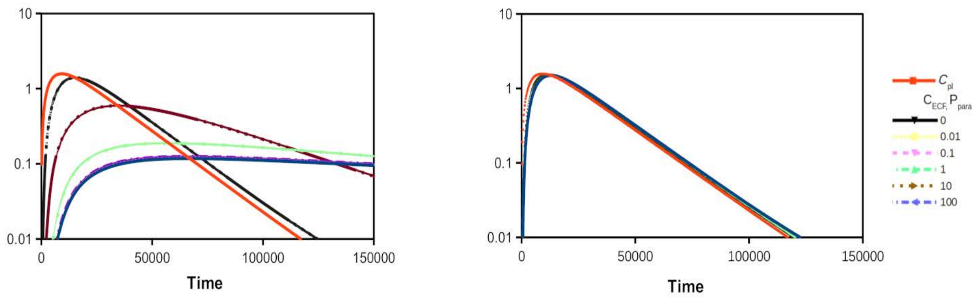

(for Eq (6)):

$$\begin{aligned}\frac{\partial u}{\partial \tau} &= 10^2 d(\frac{\partial^2 u}{\partial \xi^2} + \frac{\partial^2 u}{\partial \eta^2} + \frac{\partial^2 u}{\partial \zeta^2}) - V\frac{\partial u}{\partial \xi} \\ &- K_{1\text{on}} u(b_1^{\max} - b_1) + 10^{-2} K_{1\text{off}} b_1 \\ &- 10^{-2} K_{2\text{on}} u(b_2^{\max} - b_2) + K_{2\text{off}} b_2 \\ \frac{\partial b_1}{\partial \tau} &= K_{1\text{on}} u(b_1^{\max} - b_1) - 10^{-2} K_{1\text{off}} b_1 \\ \frac{\partial b_2}{\partial \tau} &= 10^{-2} K_{2\text{on}} u(b_2^{\max} - b_2) - K_{2\text{off}} b_2.\end{aligned}$$

The corresponding boundary conditions (Eq (10)-(11), example for Eq (10), but similar for Eq (11)) are given by:

$$d\frac{\partial u}{\partial \xi} = 10^{-3} p(w - u(\xi,\eta,\zeta,\tau)) + \frac{10^{-1} t_{\text{m}}}{s a_{\text{BBB}}(k_{\text{m}} + u(\xi,\eta,\zeta,\tau))} u(\xi,\eta,\zeta,\tau))$$

for ξ=0 and ξ=1.
The initial conditions become

$$w(\xi,\eta,\zeta,\tau = 0) = 0$$

$$u(\xi,\eta,\zeta,\tau = 0) = 0.$$
