# Supplementary material for: A 3D brain unit model to further improve prediction of local drug distribution within the brain
Source: PLoS One. 2020 Sep 23;15(9):e0238397. doi: 10.1371/journal.pone.0238397 (PMC7511021; doi:10.1371/journal.pone.0238397)
Supplement: S2 Appendix — (PDF) [file pone.0238397.s002.pdf]

S2 Appendix- The effect of paracellular permeability on PK within the brain ECF

We study the passive transcellular permeability and passive paracellular permeability separately. This is different from before, where we have studied the total passive permeability. We study the effect of paracellular transport on the PK within the 3D brain unit. The paracellular permeability can increase due to disruption of the BBB, which in turn could be a result of disease. We include paracellular permeability and study its effect on drug concentrations within the 3D brain unit. For drugs for which the passive transcellular BBB permeability is low (P_{trans}=$0.01 \cdot 10^{-7}$ μmol s^{-1}), increasing has a large impact on drug PK within the brain (Fig 1, left). For drugs with a high passive permeability, an increased paracellular permeability has less effect, as shown in Fig 1(right). Essentially, changing the paracellular permeability has a similar effect as changing the total and the transcellular permeability: both increase the transport of drug along the concentration gradient between the blood plasma in the brain capillaries and the brain ECF.
