# Supplementary material for: A 3D brain unit model to further improve prediction of local drug distribution within the brain
Source: PLoS One. 2020 Sep 23;15(9):e0238397. doi: 10.1371/journal.pone.0238397 (PMC7511021; doi:10.1371/journal.pone.0238397)
Supplement: S3 Appendix — (PDF) [file pone.0238397.s003.pdf]

S3 Appendix - The Renkin-Crone equation and the 3D brain unit model

We compare our model with the Renkin-Crone equation, which is a well-known equation relating blood flow to tissue uptake [67, 68], see Box I. The Renkin-Crone equation

S2 Fig. Determination of $C_{\mathrm{pl}}(w_1)$ and $\frac{\mathrm{d}C_{\mathrm{ECF}}}{\mathrm{dt}}(u_1)$. Time Top: Locations of w_1 and u_1, where $C_{\mathrm{pl}}(w_1)$and $\frac{\mathrm{d}C_{\mathrm{ECF}}}{\mathrm{dt}}(u_1)$ are measured, within the 3D brain unit. The black arrow indicates the direction of the brain capillary blood flow, while the green arrow indicates the direction of BBB transport. Bottom: Profiles of C_{pl} (w_1) and $\frac{\mathrm{d}C_{\mathrm{ECF}}}{\mathrm{dt}}(u_1)$ over time.

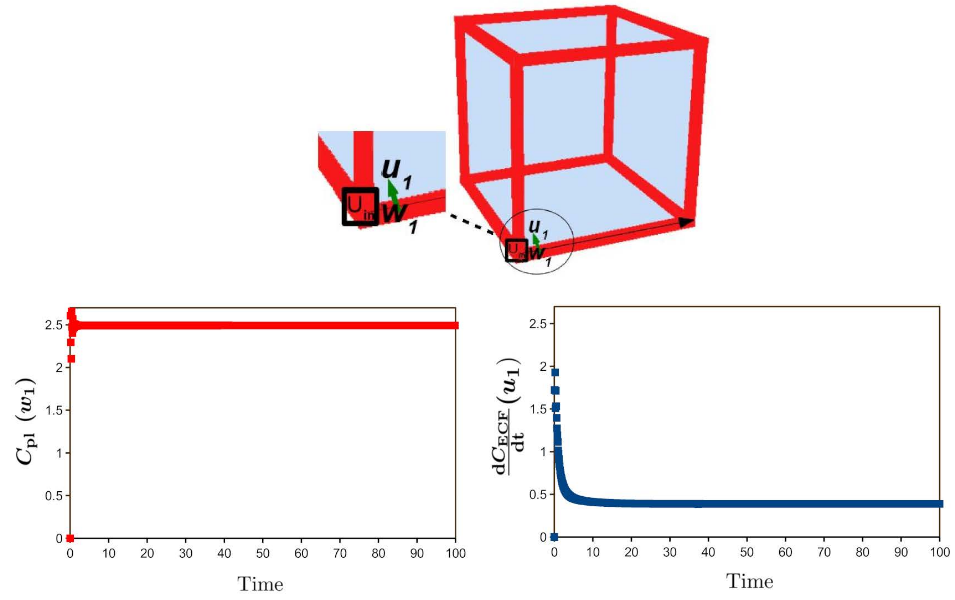

S3 Fig. The effects of v_{blood} on k_{BBB}. The effect of v_{blood} on k_{BBB} depends on P. Note that here P is taken 10^3 times its default value, see Table 2.

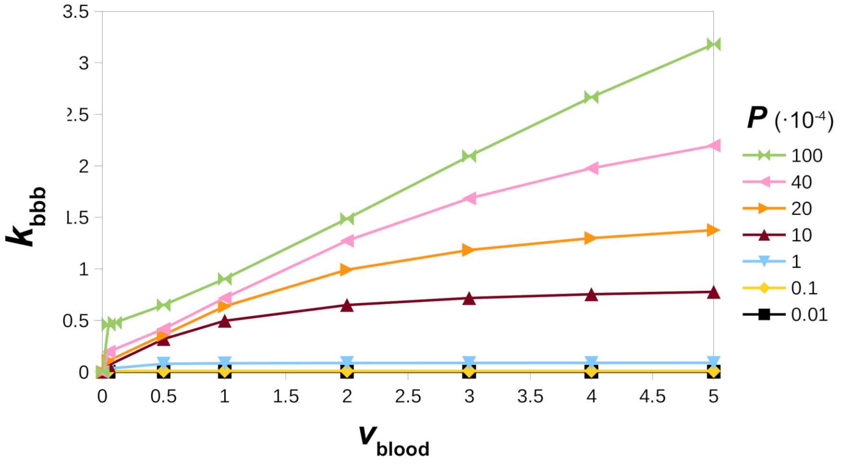

predicts that the transport of drugs across the BBB *into* the brain depends on the brain capillary blood flow rate, Q, in the presence of a large BBB permeability surface, PS. The volumetric parameters Q and PS are related to the brain capillary blood flow velocity, v_{blood}, and the BBB permeability, P, by the brain capillary and BBB surface area, SA_{cap} and SA_{BBB}, respectively. Here, we study the effect of v_{blood} on the passive transport of drug into the brain for different values of P. For this purpose, we:

1. Take a constant concentration of drug within the blood-plasma-domain (i.e. we set $C_{\text{pl}}(\text{t})=1$ for $C_{\text{pl}}(\text{t}) \in U_{\text{in}}$.
2. We simplify boundary conditions (11) and (12) to $\frac{\partial C_{\text{ECF}}}{\partial \text{x}} = P(C_{\text{pl}})$ in order to study passive *influx*, which is the passive movement of drug *into* the brain, only. Note that his is different from the approach we took previously, in which passive transport into or out of the brain ECF depends on a difference in concentration between the blood plasma and the brain ECF (see Eq(10)). Moreover, we set $T_{\text{m-in}}$=0 and $T_{\text{m-out}}$=0
3. We leave out drug binding and set $B_1{}^{\text{max}}$, $B_2{}^{\text{max}}$=0.

We measure the change in C_{ECF} ($\frac{\text{d}C_{\text{ECF}}}{\text{dt}}$) at one specific point of the 3D brain unit, (x,y,z)=($\frac{3}{2}$r,$\frac{3}{2}$r, $\frac{3}{2}$r),which we denote by u_1, as indicated in Fig 2 (top). Similarly, we measure C_{pl} at one specific point of the 3D brain unit, (x,y,z)=($\frac{3}{2}$r,$\frac{1}{2}$r, $\frac{1}{2}$r), denoted by w_1, as indicated in Fig 2 (top). It takes some time until a steady state is reached and values of C_{pl} and $\frac{\text{d}C_{\text{ECF}}}{\text{dt}}$ are approximately constant, see Fig 2 (bottom). At steady state k_{BBB}, which is the rate constant of drug transport from the blood plasma across the BBB into the brain ECF, can be determined as follows:

$$k_{\text{bbb}} = \frac{\frac{\text{d}C_{\text{ECF}}}{\text{dt}}(u_1)}{C_{\text{pl}}(w_1)} \tag{1}$$

, with $\frac{\text{d}C_{\text{ECF}}}{\text{dt}}(u_1)$ the change in C_{ECF} over time in u_1 and $C_{\text{pl}}(w_1)$ the value of C_{pl} in w_1 when both $C_{\text{pl}}(w_1)$ and $\frac{\text{d}C_{\text{ECF}}}{\text{dt}}$ (u_1) do not longer vary. Fig 3 demonstrates that the way v_{blood} affects k_{BBB} varies with the value of P. With values of P of $1{\cdot}10^{-4}$ m s^{-1} or lower, k_{BBB} is independent of v_{blood}. With values of P of $10{\cdot}10^{-4}$ m s^{-1} or higher, k_{BBB} linearly increases with v_{blood} up to a certain threshold (e.g. for P=$10{\cdot}10^{-4}$ m s^{-1}, k_{BBB} starts to approach constant levels when $v_{\text{blood}}{\geq}2$). These results correspond to the predictions of the Renkin-Crone equation (Box I).

Box I - The Renkin-Crone equation

The brain capillary blood flow affects the passive clearance of a drug across the BBB according to the Renkin-Crone equation [67,68]. The Renkin-Crone equation describes the relation between the brain capillary blood flow and transport across the BBB as follows:

$$K_{\text{in}} = QE \\ \text{with } E = 1 - e^{\frac{-PS}{Q}} \tag{2}$$

, with K_{in} the passive clearance of drug from the blood into the brain (L s^{-1}), Q (L s^{-1}) the blood flow rate in the brain capillaries and PS (L s^{-1}) the passive

permeability surface of the BBB. Both Q and PS have the same units, such that, E, the ratio of compound extracted from the blood into the brain, is dimensionless. The Renkin-Crone equation shows that the transport from the blood into the brain depends on the ratio of the BBB permeability surface (PS) and the blood flow rate (Q). When $PS \gg Q$, the extraction ratio E approaches 1, such that K_{in} is determined by changes in Q. In other words, when $PS \gg Q$, drug transport across the BBB is much faster than the rate of drug supply into the brain capillaries. Then, drug transport into the brain can only be increased by increasing Q. On the other hand, when $Q \gg PS$, E approaches 0. In this case, the drug supply into the brain capillaries is much faster than the rate of drug transport across the BBB. Then, drug transport into the brain can only be increased by increasing PS.

The Renkin-Crone equation implies that the effect of the brain capillary blood flow rate on the concentration of unbound drug exchanging with the brain is most pronounced for drugs that easily cross the BBB [68, 75], i.e. drugs for which $PS \gg Q$, or, in terms of velocity rather than rate, drugs for which $P \gg v_{\mathrm{blood}}$. Under general, non-pathological circumstances v_{blood} is around $5 \cdot 10^{-4}$ m s^{-1} (see Tables 1 and 2), which implies that BBB transport is impacted by the blood flow velocity when drug molecules have a value of P that is (much) higher than 10^{-4} m s^{-1} (i.e. 10^3 times the default value as given in Table 2).
